# Single-Bone Forearm Salvage Procedure for a Child with Acquired Radial Clubhand in a Resource Limited Centre: A Case Report

**DOI:** 10.5704/MOJ.1811.011

**Published:** 2018-11

**Authors:** H Sahdi, WH Chan, NB Dollah, A Entri

**Affiliations:** Department of Orthopaedics, Universiti Malaysia Sarawak, Kota Samarahan, Malaysia

**Keywords:** radius, hand, deformities, acquired, forearm, reconstruction procedure

## Abstract

Acquired radial clubhand deformity can be a consequence of large bone gap left by premature extensive radius osteomyelitis sequestrectomy. Single-bone forearm reconstruction is a salvage procedure when other motion-preserving techniques are not feasible. Here we present a child who developed radial clubhand deformity after an untimely sequestrectomy of radius diaphysis. In view of limited microsurgical expertise in our centre, single-bone forearm procedure was done utilising simple Kirshner wires to achieve radio-ulnar fusion. The procedure resulted in pain-free stable wrist, restoration of hand function and improved cosmesis.

## Introduction

Acquired radial clubhand deformity can be a consequence of overenthusiastic radius osteomyelitis diaphysectomy^1^. Single-bone forearm is a salvage procedure when motion-preserving method is not possible, especially for large radial defect but with intact distal radius^2^. Simple Kirschner wires can be utilised to achieve radioulnar fusion in resource-limited setting. Centres with available expertise reported successful single-bone forearm procedure with distraction osteogenesis and vascularised fibular graft methods^3, 4^.

## Case Report

A 9 year-old male child was referred to us for right radial clubhand-like deformity and limitation in performing daily activities. He was forced to modify his hand dexterity due to weak grip and pinch strength. He had previous history of extensive radius osteomyelitis that was treated by sequestrectomy. As a result of the extensive sequestrectomy, the child developed manus valgus deformity.

**Fig. 1:**
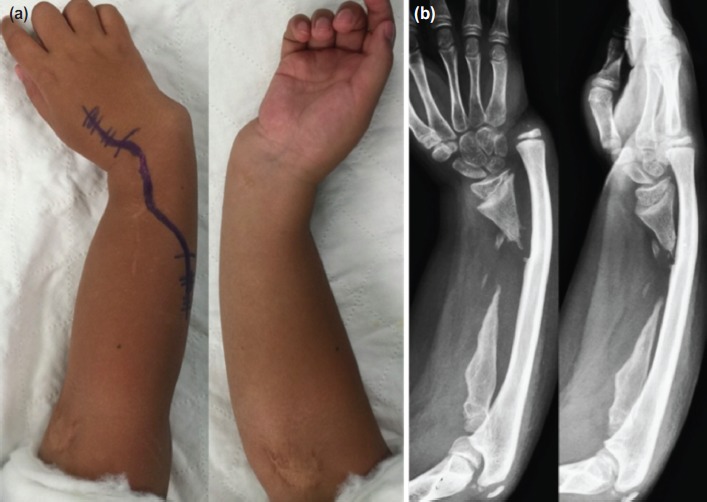
(a) Pre-operative photograph of the child showing radial clubhand deformity. (b) Radiographs showing loss of diaphysis of the right radius, intact epiphysis and hypoplastic radial head.

Examination revealed no signs of infection in the forearm. His right wrist was deviated 60 degrees radially (Fig. 1). The forearm was pronated 90 degrees. Ulnar head was prominent. Grip and wrist strength were markedly reduced. Forearm rotation was absent. Wrist dorsiflexion and palmar flexion were restricted to approximately 0 to 10 degrees. There was no neurovascular impairment. He had full range of elbow and hand movements.

Pre-operative radiographs (Fig. 1) revealed remnant distal radial epiphysis with intact distal radial physeal plate and part of the metaphysis. The distal radioulnar joint was dislocated and the radial head was hypoplastic. There was ulna overgrowth and bowing. Blood investigations including white cell count, erythrocyte sendimentation rate and C-reactive protein confirmed the absence of active infection. Taking into account the child’s extensive radial loss and the available surgical expertise in our centre, the patient was offered single-bone forearm salvage procedure.

**Fig. 2:**
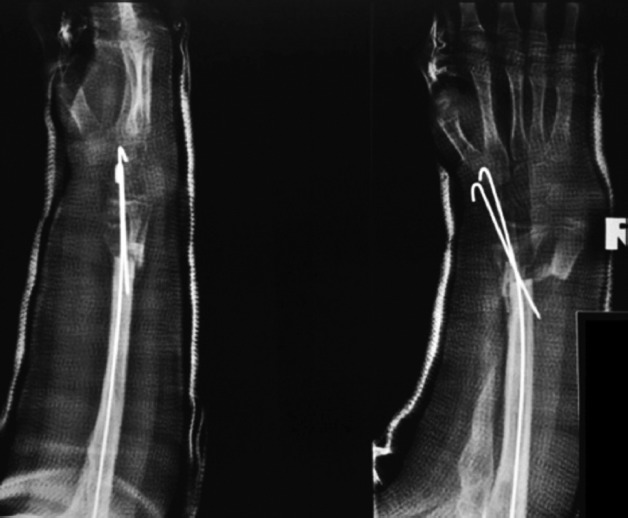
Immediate post-operative radiographs of the patient.

The patient was operated under general anaesthesia, in a supine position, with the forearm on a radiolucent table. An incision was made over the previous scar on the dorsal aspect of the forearm. The distal right radius and ulna were approached through the same incision. Radial metaphyseal end was freshened until bleeding. The right ulna was osteotomised at the level corresponding to the remaining right radius epiphysis. As there was overgrowth of the ulna, it was impossible to reduce the distal radio-ulnar joint without sacrificing 2cm of the ulna shaft. Care was also taken to avoid excessive ulna shortening, as this would cause loss of tendon tension. The distal radius was moved ulnar-wards and distally until the distal radioulnar joint was reduced. Then, the proximal segment of the ulna was radialised and the distal radius was impacted into the proximal ulna medullary cavity. Once the desired rotational alignment and length were achieved, the new distal radius-on ulnar shaft-composite was fixed with 1.6mm Kirschner wires (Fig. 2). Following wound closure, a full-length cast was applied. The Kirschner wires were removed on the 6th post-operative week.

**Fig. 3:**
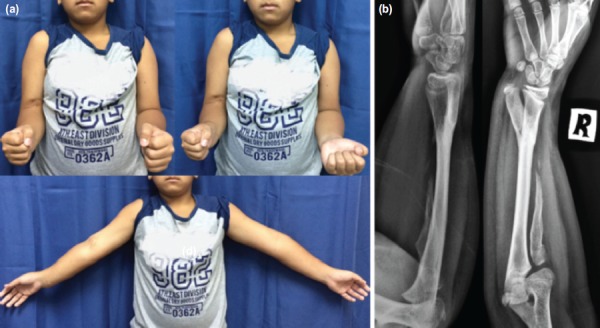
(a) Photographs of the patient at 12 months post-operatively, showing loss of right forearm rotational movement and position of upper limbs with elbow in extension. (b) Forearm radiograph showing solid union at the fusion site.

At 12 months post-surgery, the child had normal use of his right hand and was pleased with the cosmetic appearance. The wrist was pain free with full range of motion. There was no forearm rotation (Fig. 3) but the child had learned to overcome this by compensatory shoulder rotation. Radiographs revealed solid union at the fusion site. The distal ulna had developed osseous bridge with the adjacent proximal ulna, creating a Y-shaped single bone forearm. Long-term follow-up is needed to monitor for recurrence of the deformity and limb length discrepancy.

## Discussion

Overzealous resection of sequestrum will lead to severe radial shortening, relative ulna overgrowth and angulation, and radial clubhand-like deformity. Subsequently, the deformity will result in handgrip weakness, wrist pain and cosmetic disfigurement^1^. The options of treatment depend on the remaining part of the diaphysis. For large radial defect but with undamaged distal radial epiphysis, like in our case, Rasool recommended the single-bone forearm technique^1^.

Single-bone forearm was first described by Hey Groves for traumatic radius diaphyseal bone loss in an adult^2^. The single-bone forearm is based on the fundamental principle of the “ulna makes the elbow and radius makes the wrist”. To ensure favourable outcome, a normal hand with intact wrist and elbow joints is required pre-operatively^5^.

The final position of radioulnar fusion should be determined on a case-by-case basis, taking into consideration age, dexterity, occupation, hobbies and patient’s preference. Most authors recommended a position halfway between pronation and supination or in slight pronation^2, 5^. Unfortunately, single-bone forearm will sacrifice forearm rotation. Therefore, single-bone forearm should only be reserved when bone loss is too extensive for other reconstructive techniques^1, 5.^

We decided to perform single-bone forearm with simple Kirschner wires in this patient, as there was limited microsurgical expertise in our centre. The distal radial stump was too short for adequate screw or pin placement, and therefore, unsuitable for usage of plate and screws or external fixator.

Another method to construct single-bone forearm is by distraction osteogenesis^3^. Distraction osteogenenesis technique with circular external fixator involves complex instrumentation. This method requires high commitment from patients with regards to prolonged period of treatment and meticulous pin care. Furthermore, external fixator method has other complications such as pin site problems, nerve palsies, malunion, re-fracture and infection^1^. The use of vascularised bone graft is technically demanding. Arai *et al* reported the use of vascularised fibular graft to create single-bone forearm^4^. However, fibular graft can be complicated with infection, graft resorption, refracture, delayed union and donor site morbidity^1, 4^.

Single-bone forearm is a salvage procedure for cases where the usual reconstruction techniques are not feasible. To date, there is no consensus on the standard indication for this technique and the ideal method for achieving single-bone forearm. Selection of method must be made on an individual basis, depending on the available surgical expertise, amount and area of bone loss, soft tissue condition, patient’s age, compliance, daily activities and personal expectations.

In resource-limited centres, Kirschner wires can be utilised to achieve single-bone forearm. This method is simple, requires minimal technology and practical for patients with compliance issues. It also negates the need to sacrifice another healthy site for harvesting bone graft, thus avoiding donor site morbidity.

## Conflict of Interest

The authors declare no conflicts of interest.
